# Greater large conducting airway luminal area in adult patients with interstitial lung disease

**DOI:** 10.14814/phy2.70578

**Published:** 2025-09-24

**Authors:** Alex J. Miller, Erik A. Ovrom, Solomiia Zaremba, Jonathon W. Senefeld, Chad C. Wiggins, Paolo B. Dominelli, Juan G. Ripoll, Brian T. Welch, Michael J. Joyner, Andrew H. Ramsook

**Affiliations:** ^1^ Department of Anesthesiology and Perioperative Medicine Mayo Clinic Rochester Minnesota USA; ^2^ Alix School of Medicine Mayo Clinic Rochester Minnesota USA; ^3^ Department of Physiology and Biomedical Engineering Mayo Clinic Rochester Minnesota USA; ^4^ Department of Health and Kinesiology University of Illinois at Urbana‐Champaign Urbana Illinois USA; ^5^ Beckman Institute for Advanced Science and Technology University of Illinois at Urbana‐Champaign Urbana Illinois USA; ^6^ Department of Kinesiology Michigan State University East Lansing Michigan USA; ^7^ Department of Kinesiology and Health Sciences University of Waterloo Waterloo Ontario Canada; ^8^ Faculty of Kinesiology University of Calgary Calgary Alberta Canada; ^9^ Division of Critical Care Medicine Mayo Clinic Rochester Minnesota USA; ^10^ Department of Radiology Mayo Clinic Rochester Minnesota USA

**Keywords:** airway morphology, airway remodeling, computer tomography, dysanapsis, pulmonary fibrosis, sex differences

## Abstract

Interstitial lung disease (ILD) encompasses pulmonary disorders characterized by chronic inflammation and fibrosis that disrupt pulmonary gas exchange. While ILD‐related changes in the lung parenchyma are well‐documented, less is known about how ILD affects luminal area within the large conducting airways. This retrospective, case–control study tested the hypothesis that patients with ILD would have greater large conducting airway luminal areas than healthy matched controls. Three‐dimensional reconstructions of computed tomography images were used to quantify airway luminal areas in patients with ILD (*n* = 82; 54% female) and healthy controls matched for age, sex, and height. Patients with ILD had 16%–22% greater airway luminal areas across all seven measured large conducting airways compared to controls (all *p* < 0.001). Among patients with ILD, males had 17%–34% greater height‐normalized airway luminal areas than females (all *p* < 0.05). These data provide evidence that ILD is associated with greater large conducting airway luminal area, even when matched for key demographic factors. Consistent with observations in health, males with ILD exhibited greater height‐normalized airway size than females. These findings offer new insight into airway remodeling in ILD and highlight potential sex differences in airway luminal area.

## 1 INTRODUCTION

Interstitial lung disease (ILD) encompasses a family of restrictive lung diseases characterized by chronic inflammation and resultant fibrosis (Antoniou et al., 2014; Travis et al., 2013). ILD is associated with damage to the interstitial bed and alveoli, which synergistically disrupts pulmonary gas exchange (Agustí et al., 1991; Lama et al., 2003). While pulmonary fibrosis in the lung interstitium of patients with ILD is well‐documented (Becker et al., 2008; Frankel et al., 2004; Maher et al., 2007; Margaritopoulos et al., 2012), less is known about how this disease may affect conducting airway luminal area.

Previous investigations examining the effect of ILD on airway luminal area have primarily focused on small (7–17 generations) airways (Adams et al., 2020; Ikezoe et al., 2021; Verleden et al., 2020). The small airways, often referred to as “the quiet zone” (Mead, 1970), are conceptually considered a respiratory region where ILD may progress without detectable clinical signs and symptoms. Studies have indicated that patients with ILD develop honeycomb cysts in lungs affected by idiopathic pulmonary fibrosis and exhibit larger small airway luminal areas (Ikezoe et al., 2021). Many of these studies have been limited to using preclinical rodent models of ILD, explanted human pulmonary tissue, or clinical studies with small sample sizes. Limited investigations have examined the impact of ILD on in vivo human large conducting airways. Recent evidence demonstrates that males with ILD may have larger conducting airways than controls, highlighting the relevance of investigating airway morphology in ILD patients (Maetani et al., 2024). Additionally, previous work has linked larger central airway size to clinical prognosis in ILD (Handa et al., 2022), further the supporting the importance of understanding airway morphology in this population. The present study builds on this emerging evidence by examining both males and females and exploring potential sex differences in airway size among patients with ILD. In this framework, these large conducting airway luminal area measurements may provide a more comprehensive understanding of the pathophysiological effects associated with ILD.

Accordingly, the primary objective of our study was to determine the relationship between ILD and airway luminal area by comparing in vivo large conducting airway luminal area between patients with ILD and control subjects. This retrospective, case–control study used chest computed tomography (CT) scans to test the hypothesis that patients with ILD would have greater conducting airway luminal area than healthy control subjects matched for age, sex, and height. Additionally, because there are well‐established sex differences in airway luminal area across the lifespan (Dominelli et al., 2018; Ripoll et al., 2020) and the broader importance of considering sex as a biological variable (Clayton & Collins, 2014), we included data for both males and females to assess potential sex differences in airway luminal area among patients with ILD.

## 2 MATERIALS AND METHODS

### 2.1 Ethical approval

This retrospective study was approved by the Mayo Clinic Institutional Review Board (IRB #17‐008537) and adhered to the standards set forth in the *Declaration of Helsinki*, except registration in a database. Routine clinical care resulted in CT scans of the thorax. Informed consent was waived as no identifiers were used in analyses, these clinical data already existed, the research did not affect patient care, and neither the patients nor their legal guardians opted out of their data being used for research. Consent waiver was approved by the Mayo Clinic Institutional Review Board.

### 2.2 Patients

#### 2.2.1 ILD patients

Initial inclusion criteria for ILD patients were (1) diagnosis by a pulmonologist, (2) at least one thoracic CT scan following their diagnosis, and (3) pulmonary function test (PFT) data. Exclusion criteria for patients included: less than 18 years of age, any previous surgical intervention to the lungs, end‐stage liver disease, neoplasm, Sjögren's syndrome, and additional cardiopulmonary disease (e.g., chronic obstructive pulmonary disease, asthma, heart failure, pleural effusion, obstructive sleep apnea, acute coronary syndrome, and pulmonary infection). Listed conditions were excluded as they may have confounding effects on airway anatomy and pulmonary function. Exclusion criteria for CT scans included: poor scan resolution and incompatibility with the software used to measure airway luminal area. CT scans were selected on the basis of (1) scan resolution and (2) temporal proximity between CT scan date and PFT date. CT scans were analyzed for large conducting airway cross‐sectional luminal area. All ILD diagnoses were made by board‐certified pulmonologists, following standard diagnostic practices that included clinical history, imaging, and pulmonary function testing.

#### 2.2.2 Control subjects

The initial inclusion criterion for control subjects was a chest CT scan performed to evaluate suspected pulmonary embolism in adults aged 18 years or older. All individuals included in this study were confirmed to be negative for pulmonary embolism. At the time of this retrospective study, patient medical histories were independently reviewed by a physician, confirming no evidence of pulmonary disease. Exclusion criteria were consistent with those used for ILD patients and additionally included idiopathic pulmonary fibrosis, tobacco use, end‐stage kidney disease, ascites, and class III obesity (body mass index ≥40 kg·m^−2^) as these conditions may have confounding effects on airway anatomy and pulmonary function.

#### 2.2.3 Patient matching

Using a nearest neighbor matching algorithm, potential control subjects and ILD patients were sequentially, individually matched (1:1) for sex, age, and height (Figure S1).

### 2.3 Image acquisition

As described previously (Dominelli et al., 2018; Guo et al., 2020; Jeltema et al., 2022; Ripoll et al., 2020), institutionally standardized CT algorithms are used for chest imaging at local medical centers. A posterior–anterior and lateral topogram is obtained at 120 kV and 35 mA. Spiral acquisitions with a pitch of 1.2 are utilized. Kilovoltage is set at 120 with a standard milliampere‐second value of 140. Images are acquired at end‐inspiration. Post imaging reconstructions are obtained in the axial and coronal planes using a B46 kernel. Slice thicknesses of 1.5 mm and 3.0 mm are reconstructed. Maximal intensity projections in the axial and coronal planes are completed with a slice thickness of 10 mm and reconstruction increment of 2.5 mm. Participants inspired before imaging was performed. Because participants are not instructed to inhale maximally to total lung capacity, we were unable to match lung volumes (see Section 4.4 below). Using image analysis, we computed lung volume and expressed it as a percent of total lung capacity for patients with ILD and as a percent of predicted total lung capacity for control subjects.

### 2.4 Airway measurements

**FIGURE 1:**
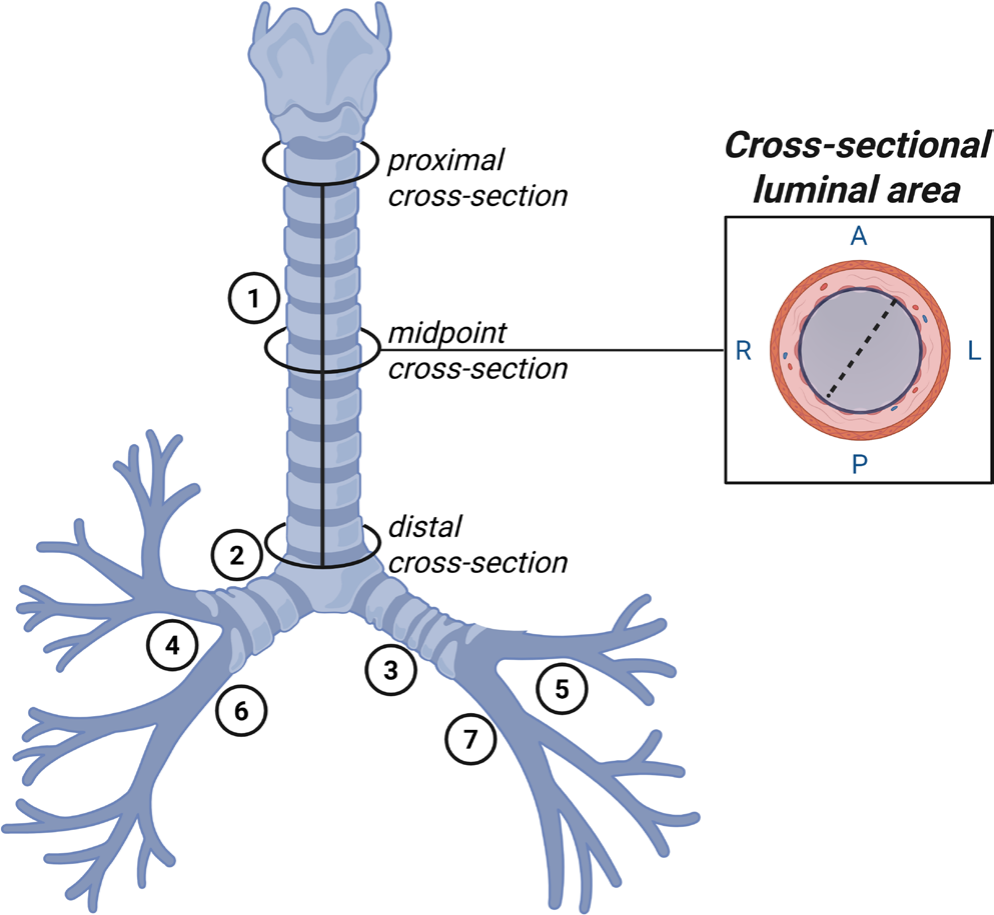
Graphical representation of the large conducting airway tree. Circles represent specific locations at which the cross‐sections of the trachea (Antoniou et al., 2014) were taken. Other numbers represent additional airway segments measured. The dotted line within the inset represents a diameter measurement. 2, right main bronchus; 3, left main bronchus; 4, right upper lobe; 5, left upper lobe; 6, bronchus intermediate; 7, left lower lobe; A, anterior; L, left; P, posterior; R, right. Created with BioRender (https://biorender.com).

Images were analyzed using commercially available software (TeraRecon, AQI, Foster City, CA, USA) as described previously (Dominelli et al., 2018; Guo et al., 2020; Jeltema et al., 2022; Ripoll et al., 2020). After visual isolation from surrounding tissue, the software created a three‐dimensional reconstruction from CT scans of the lungs and the airways. The three‐dimensional reconstruction and optimized visualization enabled precision estimation of both absolute lung volume and cross‐sectional luminal area of the large conducting airways. Airway measurements were all performed by a single individual (AJM). A subsection of measurements was validated against independent training samples used in the laboratory and repeated by multiple investigators (SZ and AHR). The cross‐sectional areas of conducting airway segments were digitally measured at three points (proximal, middle, and distal points) for each of the following airway segments: the trachea, right and left main bronchus, right and left upper lobes, bronchus intermediate, and left lower lobe. The proximal cross‐section of the trachea was defined as the point below the cricoid cartilage. The distal cross‐section was defined as the point above its bifurcations. Anatomical bifurcations defined the proximal and distal points of the measured bronchi. The midpoint was defined as half the length of the airway segment. A graphical representation of an airway tree describing cross‐sectional area measurements is displayed in Figure 1. For the analysis of sex differences in ILD patients, airway luminal areas were represented relative to patient height (“height‐normalized”) using the quotient of airway luminal area (mm^2^) to patient height (cm).

### 2.5 Pulmonary function testing (PFT)

Institutional standards of clinical care for patients with ILD include routine PFTs. Thus, when available, PFT data (total lung capacity [TLC], computed lung volume/TLC, ratio of forced expired volume in 1 s to forced vital capacity [FEV_1_/FVC], and diffusing capacity for carbon monoxide [D_LCO_]) were abstracted from medical records of patients with ILD. CT scans were selected to optimize temporal proximity relative to PFT collection. Specifically, the median time between PFT and CT was 11.5 days, indicating that for most participants, the PFT testing occurred within about 2 weeks of the CT scan. PFT data were not available in the control cohort; thus, predicted values were used for the purpose of comparison.

### 2.6 Statistical analysis

Normality was assessed using Shapiro–Wilk tests, and homoscedasticity was assessed using Levene's test. Statistical models were determined based on normality and homoscedasticity. Univariate analyses of variance (ANOVA) were used to compare participant characteristics (age, height, mass, body mass index, days since diagnosis, and smoking history) and pulmonary characteristics (computed lung volume, TLC, computed lung volume/TLC, FEV_1_/FVC, and D_LCO_) between patients with ILD and matched control subjects. ANOVAs, separated by airway segment, were used to compare metrics of conducting airway luminal area between patients with ILD and matched control subjects. One‐way ANOVA statistical models were performed using a representation of airway luminal area—the measurement of the average of three cross‐sectional areas (proximal, middle, and distal). Descriptive statistics are presented as mean ± standard deviation (SD) within the text and tables for normally distributed data and are presented as median (IQR) for non‐normally distributed data. *p* values were reported, and the interpretation of findings was based on *p* < 0.05. Analyses were performed with the use of IBM Statistical Package for Social Sciences version 28 statistical package (Armonk, New York, USA).

Notably, several distributions of large conducting airway luminal cross‐sectional area failed tests of normality. Tukey's fences were used to identify statistical outliers. Tukey's methods identify values that are 1.5 IQR below Q1 and 1.5 IQR above Q3 as outliers, where IQR denotes interquartile range, Q1 denotes lower quartile, and Q3 denotes upper quartile. In this context, outliers approximate the 1st or 99th percentile of a dataset. After removal of outliers, tests of normality were performed again. If sample distributions failed tests of normality after removal of outliers, Kruskal–Wallis tests were used. If tests of normality were passed after the removal of outliers, univariate ANOVA tests were performed on the dataset sans outliers. Among males, five airways were normally distributed (trachea, right main bronchus, bronchus intermediate, right upper lobe, and lower main bronchus) and two airways failed tests of normality (left lower lobe and left upper lobe). However, after removing outliers the left upper lobe no longer failed tests of normality; thus, parametric tests were used for statistical analyses. The left lower lobe continued to fail tests of normality; thus, nonparametric tests were used for statistical analyses. Among females, three airways were normally distributed (bronchus intermediate, right upper lobe, and lower main bronchus) and four airways failed tests of normality (trachea, right main bronchus, left lower lobe, and left upper lobe). However, after removing outliers, the trachea, right main bronchus, and left lower lobe no longer failed tests of normality. The left upper lobe continued to fail tests of normality; thus, nonparametric tests were used for statistical analyses.

## 3 RESULTS

### 3.1 Screening

#### 3.1.1 ILD patients

Medical histories of 804 patients with ILD who met the initial search criteria were screened by the research team. After screening, CT scans of 163 patients with ILD who met the inclusion criteria were screened for potential assessment of airway luminal area. During data abstraction, 81 additional patients with ILD were excluded due to incomplete PFT data (*N* = 72), CT scans performed before ILD diagnosis (*N* = 7), or poor CT scan quality (defined by inability to aptly visualize one or more of the large conducting airways, *N* = 2). The final cohort of patients with ILD analyzed in this study represented 82 patients, including 38 males and 44 females.

#### 3.1.2 Control subjects and patient matching

Medical histories of 2034 participants who met the initial search criteria were screened. After exclusion, 132 participants were considered for matching, and their CT scans were analyzed for large conducting cross‐sectional luminal area. Using our nearest neighbor matching algorithm, another 50 participants were excluded due to inadequate demographic and anthropometric characteristics for matching.

**FIGURE 2:**
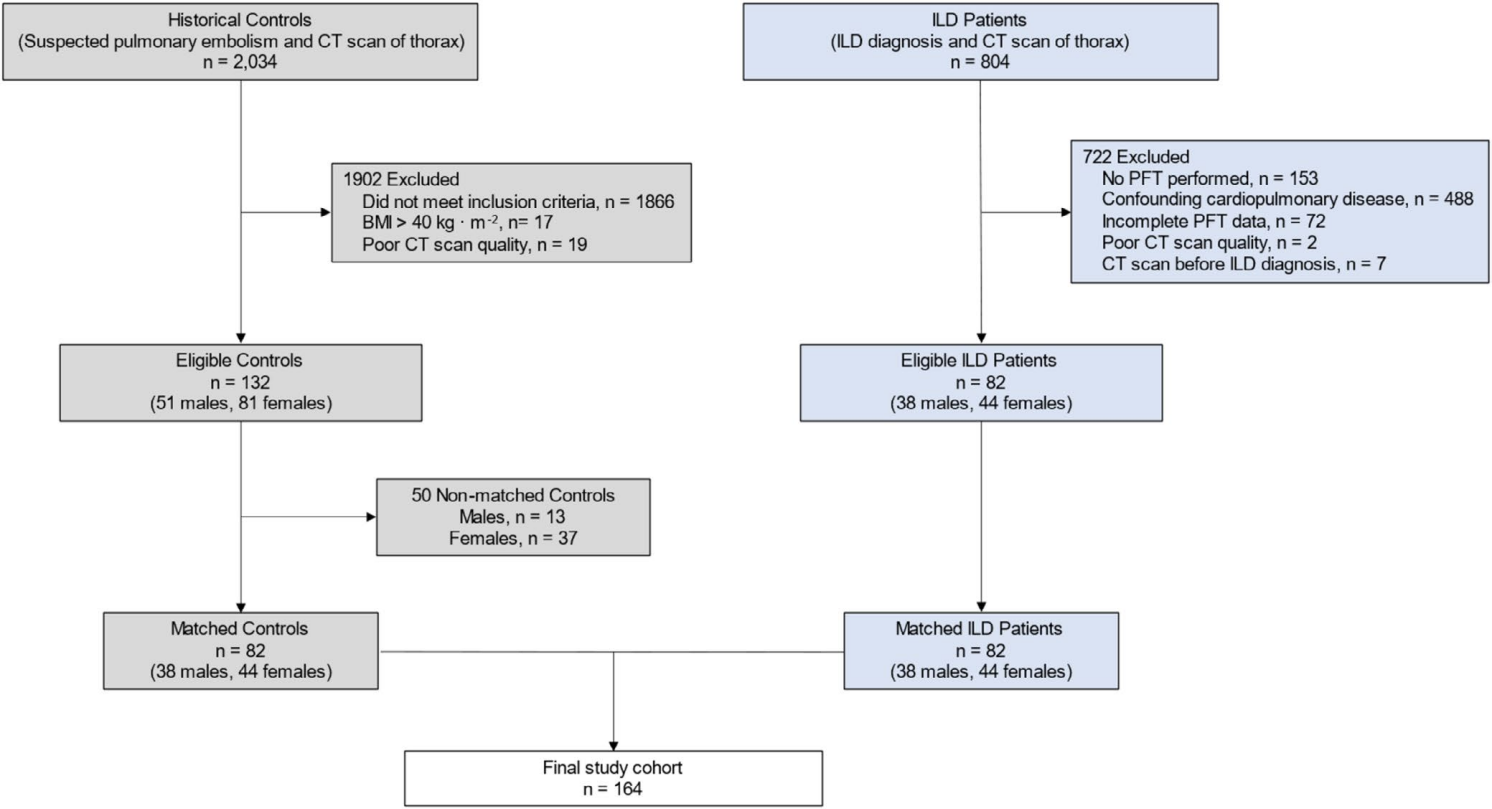
Participant inclusion paradigm of patient eligibility and control subject matching for the study. BMI, body mass index; CT, computed tomography; ILD, interstitial lung disease; PFT, pulmonary function test.

For the control cohort, 132 participants (51 males and 81 females) who were included in our previous study (Dominelli et al., 2018) were considered for inclusion. Using a nearest neighbor matching algorithm, potential control subjects were individually (1:1) matched for sex, age, and height (Figure S1). In this framework, the final cohort of control subjects was analogous to the ILD patients and included 82 participants (38 males and 44 females). The participants' inclusion paradigm is displayed in Figure 2.

### 3.2 Patient characteristics

Patient characteristics are displayed in Table 1. Control subjects had a lower computed lung volume relative to predicted TLC compared to patients with ILD (*p* < 0.05). Among males, there were no group differences (ILD vs. control subjects) in mass, body mass index (BMI), and computed lung volume. However, females with ILD had smaller mass and BMI compared to female control subjects (*p* < 0.05). Females with ILD also had larger computed lung volume (*p* < 0.05) compared to female control subjects. Subgroups of specific ILD diagnoses are presented in Table S2.

**TABLE 1:** Patient characteristics.

Variable	Males			Females		
	ILD	Control	*p* Value	ILD	Control	*p* Value
Cohort size, *n*	38	38	–	44	44	–
Participant characteristics						
Age, years	62 ± 10	60 ± 13	0.459	61 ± 14	61 ± 14	0.796
Height, cm	177 ± 5	178 ± 7	0.350	162 ± 7	163 ± 6	0.379
Weight, kg	92 ± 15	95 ± 14	0.383	74 ± 17	90 ± 24	**<0.001**
BMI, kg·m^−2^	30 ± 4	30 ± 5	0.537	28 ± 6	34 ± 9	**<0.001**
Days since diagnosis	495 ± 1003	–	–	366 ± 801	–	–
Smoking history, pack/years	23 ± 30	–	–	7 ± 15	–	–
Scan and PFT						
Computed lung volume, cm^3^	4106 ± 1331	4171 ± 1548	0.848	3266 ± 1030	2822 ± 729	**0.023**
Computed lung volume/TLC, %	85 ± 17	58 ± 21	**<0.001**	83 ± 18	55 ± 13	**<0.001**
TLC, liters	4.7 ± 1.6	7.3 ± 0.7^a^	**<0.001**	3.8 ± 1.0	5.2 ± 0.4^a^	**<0.001**
TLC, % predicted	71 ± 21	–	–	70 ± 19	–	–
FEV_1_/FVC	0.79 ± 0.1	0.78 ± 0.02^a^	0.244	0.77 ± 0.1	0.79 ± 0.02^a^	0.291
FEV_1_/FVC, % predicted	101 ± 9	–	–	97 ± 17	–	–
D_LCO_, mL_CO_/sec/mmHg	15 ± 7	–	–	12 ± 4	–	–
D_LCO_, % predicted	61 ± 26	–	–	57 ± 17	–	–

*Note*: Data are reported as count or mean ± standard deviation (SD). Data are compared using separate univariate ANOVAs. *p* Values are reported for between group comparisons (ILD vs. control subjects) for males and females separately. “–” indicates no data available. Bold was used to indicate *p* < 0.05 values.

Abbreviations: BMI, body mass index; D_LCO_, diffusing capacity of the lungs for carbon monoxide; FEV_1_/FVC, forced expiratory volume in the first 1 s over forced vital capacity; TLC, total lung capacity.

^a^ Predicted values used as no other data were available.

### 3.3 Airway luminal area: ILD versus health

**FIGURE 3:**
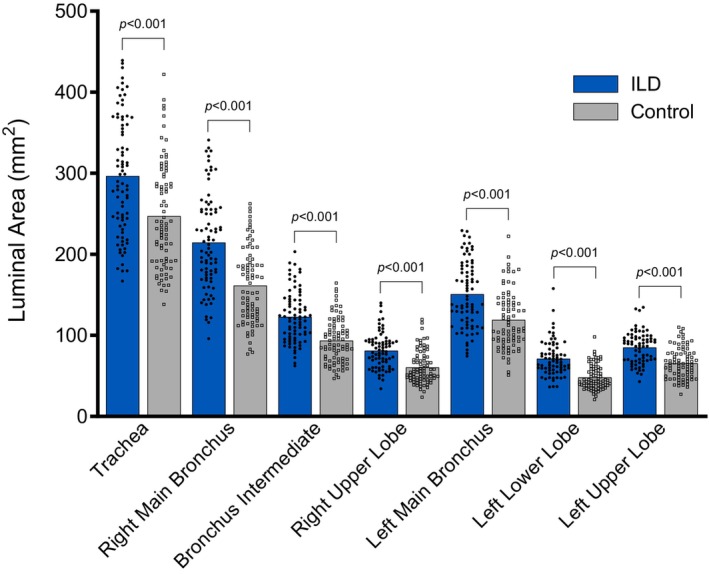
Luminal airway area of patients with ILD and healthy, sex‐, height‐, and age‐matched control subjects. Specific *p* values for group comparisons are displayed. Data are presented as mean with individual data. Outlier data are excluded.

In all measured large conducting airways, average luminal area was greater among patients with ILD compared to control subjects (Figure 3; all *p* < 0.001). Depending on the airway segment, cross‐sectional luminal area was 20%–48% greater in ILD patients compared to control subjects (Table 2).

**TABLE 2:** Airway luminal area of males and females diagnosed with ILD and a height‐ and age‐matched control cohort.

Airway luminal area (mm^2^)	Males			Females		
	ILD	Control	*p* Value	ILD	Control	*p* Value
Trachea	356 ± 49	300 ± 50	<0.001	245 ± 46	202 ± 37	<0.001
Right main bronchus	256 ± 45	199 ± 33	<0.001	179 ± 44	129 ± 28	<0.001
Bronchus intermediate	144 ± 28	114 ± 22	<0.001	104 ± 22	76 ± 17	<0.001
Right upper lobe	95 ± 19	73 ± 20	<0.001	70 ± 16	50 ± 13	<0.001
Left main bronchus	179 ± 32	146 ± 29	<0.001	125 ± 30	96 ± 21	<0.001
Left upper lobe	98 ± 17	80 ± 23	<0.001	77 ± 19	57 ± 16	<0.001
Left lower lobe	77 (64–90)	55 (42–69)	<0.001	61 (53–70)	40 (35–46)	<0.001

*Note*: Data are reported as mean ± standard deviation (SD) for normally distributed data and are reported at median (IQR) for non‐normally distributed data. Data are compared using separate univariate ANOVAs for normally distributed data and are compared using Kruskal–Wallis tests for non‐normally distributed data. *p* Values are reported for between group comparisons (ILD vs. control) for males and females separately.

### 3.4 Sex‐related differences in ILD

**FIGURE 4:**
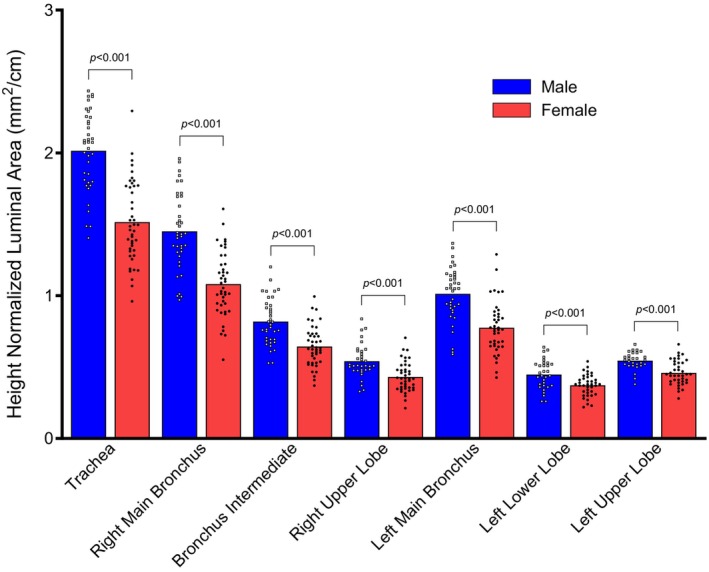
Luminal airway area normalized to height in males and females with ILD. Specific *p* values for sex differences are displayed. Data are presented as mean with individual data. Outlier data are excluded.

In all measured large conducting airways, height‐normalized luminal area was greater in males with ILD compared to females with ILD, Figure 4 (*p* < 0.001). Height‐normalized luminal area of males with ILD was 19%–34% greater depending on the airway segment than females with ILD (Table 3).

**TABLE 3:** Airway luminal area (mm^2^) normalized to height (cm) of males and females diagnosed with ILD.

Height normalized airway luminal area (mm^2^/cm)	ILD		
	Males	Females	*p* Value
Trachea	2.02 ± 0.28	1.51 ± 0.29	**<0.001**
Right main bronchus	1.45 ± 0.27	1.08 ± 0.23	**<0.001**
Bronchus intermediate	0.82 ± 0.16	0.64 ± 0.14	**<0.001**
Right upper lobe	0.54 ± 0.12	0.43 ± 0.10	**<0.001**
Left main bronchus	1.01 ± 0.19	0.78 ± 0.18	**<0.001**
Left upper lobe	0.54 ± 0.06	0.46 ± 0.09	**<0.001**
Left lower lobe	0.45 ± 0.10	0.37 ± 0.08	**<0.001**

*Note*: Data are reported as mean ± standard deviation (SD). Data are compared using separate univariate ANOVAs. *p* values are reported for intragroup sex differences (males with ILD vs. females with ILD). Bold was used to indicate *p* < 0.05 values.

## 4 DISCUSSION

The primary aim of this study was to evaluate large conducting airway luminal area among patients with ILD compared to control subjects matched for age, sex, and height. In support of our primary hypothesis, we found that patients with ILD had greater cross‐sectional luminal areas compared to matched control subjects. This observation is consistent with recent reports (Maetani et al., 2024) suggesting that increased proximal airway size may be a characteristic of ILD. Our findings extend this emerging body of literature by including both males and females and further examining potential sex differences in conducting airway morphology. Exploratory analyses demonstrated that the expected sex difference persisted in patients with ILD, resulting in males having greater cross‐sectional luminal areas in the large conducting airways than females. Notably, this observed sex difference in large conducting airway luminal area is also commonly observed in healthy youths (Ripoll et al., 2020) and adults (Dominelli et al., 2018; Mann et al., 2021).

### 4.1 Pathophysiology of ILD in the conducting airways

Previous research has focused on the effects of fibrosis on surfaces of the lungs responsible for gas exchange (Caminati et al., 2013; Corte et al., 2012; Margaritopoulos et al., 2010; Rice et al., 2003). Findings suggest that ILD is associated with an increase in the diameter of small airways (Ikezoe et al., 2021; Verleden et al., 2020). As the lungs become inflamed and consequently fibrosed throughout the progression of ILD, compliance (Orens et al., 1995; Radwan et al., 1999; Zielonka et al., 2010) decreases, thereby causing a restrictive breathing pattern and ineffective ventilation (Cortes‐Telles et al., 1900; Hamada et al., 2007; Wells et al., 1997). In this context, a decrease in ventilation due to airway dilation and a resultant increase in anatomic dead space (Plantier et al., 2018) may compound respiratory dysfunction.

One potential explanation for our findings is progressive fibrosis extending from the parenchyma into the conducting airways. Ikezoe et al. investigated fibrosis in the small conducting airways and discovered concomitant increases in markers of fibrosis and increases in luminal area (Ikezoe et al., 2021). Pulmonary fibrosis in the large conducting airways leads to reduced compliance (Zielonka et al., 2010) and elasticity, rendering the conducting airways dilated and rigid. Coupling this pathophysiological process with concomitant decreases in compliance of the surrounding pulmonary tissue (Radwan et al., 1999) leads to an increased metabolic cost of breathing (Molgat‐Seon et al., 2019). This framework is supported by our findings as airway luminal area was greater in patients with ILD, which may indicate fibrotic tissue in the large conducting airways and may help explain increased work of breathing (Phillips et al., 2012), found in an animal model, and dyspnea in ILD (Collard et al., 2003; Schaeffer et al., 2019). Overall, in the context of previous studies, our findings suggest that ILD disrupts the lungs more extensively than what has been thought to be the disease's primary target, the bronchovascular interstitium.

### 4.2 Sex differences in airway anatomy

We found that among patients with ILD, males had greater luminal areas normalized to height in the large conducting airways than females. Limited research has been performed to examine potential biological sex differences in airway diameter among patients with ILD (Coxson et al., 1997; Figueira de Mello et al., 2010; Ikezoe et al., 2021; Tanabe et al., 2020; Verleden et al., 2020), although sex differences in airway luminal area are commonly observed in healthy control subjects across the lifespan, even when adjusted for factors that commonly affect airway size, like height. Epidemiological data have shown that males with ILD have lower survival rates, increased prevalence of disease, and attenuated lung function (Han et al., 2008; Salisbury et al., 2016; Sauleda et al., 2018). Such findings warrant investigation into the role of sex‐specific hormones in pulmonary fibrosis; however, the effect of biological sex hormones on pulmonary fibrosis and airway remodeling remains unclear (Gharaee‐Kermani et al., 2005; Redente et al., 2011; Tofovic et al., 2009; Voltz et al., 2008). Previous studies examining sex differences in airway luminal area among patients with ILD have been limited to mice models, using ex vivo human lungs, lacking a comparator control group, and/or evaluating only the small airways. Our research aimed to address some of these gaps in the literature.

### 4.3 Potential clinical implications

As pulmonary fibrosis progresses, compliance of the conducting airways and parenchyma is reduced (Sansores et al., 1996). Thus, lung volumes are decreased. In this framework, pulmonary fibrosis in the interstitial space results in a compounding effect of conducting airway dysfunction and a reduction in gas exchanging tissue, leading to an even greater reduction of the diffusive capabilities of the lungs. In addition to the known effects of reduced lung compliance, our findings suggest that larger proximal airway size may also contribute to the preserved or elevated FEV_1_/FVC ratio commonly observed in ILD through reduced airway resistance.

Future work should investigate the mechanisms behind our findings of greater large conducting airway luminal area. Characterizing potential histopathological changes in the large conducting airways may explain our findings of increased luminal area. If markers of fibrosis and inflammation are found in the large conducting airways of patients with ILD, comprehensive treatment of ILD might include interventions to not only target the sequelae of inflammation and fibrosis in the parenchyma but also dysfunction and remodeling within the conducting airways (McParland et al., 2003). However, in order to systematically treat the downstream effects of ILD such as dyspnea, we must comprehensively understand the effect of the disease not only in the parenchyma but also in the small and large conducting airways.

### 4.4 Limitations

Several limitations resulted from the design of this retrospective study. First, CT‐derived lung volumes were not standardized to total lung capacity, as participants were only instructed to inspire and hold their breath. This variability in lung inflation, particularly among control subjects, is common in retrospective CT datasets and likely contributed to differences in lung volume, including the unexpected finding of larger computed lung volumes in females with ILD compared to female controls. Notably, lung volume is likely to have the greatest influence on more distal airway compared to first and second generation airways (Kambara et al., 2014). In this context, this limitation likely does not affect our primary findings on large conducting airways. Second, BMI was higher in female control subjects than in female ILD participants; however, supplementary analyses demonstrated that BMI was not correlated with airway luminal areas in females, suggesting that BMI did not confound our primary findings. Third, our methodology and study design only allow for observational conclusions. Our findings suggest an association between ILD and greater airway luminal area but do not infer a causal relationship. Fourth, PFT data were not available for healthy control subjects, and predicted values were calculated and used for the purpose of group comparisons. Fifth, the CT image acquisition used larger slice thickness reconstructions (1.5 mm and 3.0 mm). Thinner slice reconstructions of less than 1.5 mm may provide improved precision, particularly for smaller or more distal airways. However, our results closely align with other literature, suggesting that this limitation did not meaningfully impact the interpretation of our findings.

## 5 CONCLUSIONS

These data provide evidence that patients with ILD have significantly greater cross‐sectional airway luminal area than matched control subjects. We also conclude that males with ILD have significantly greater cross‐sectional airway area normalized to height than females with ILD, suggesting absent sex differences in large conducting airway luminal area changes. Overall, these findings may, in part, explain the symptomatology of ILD, such as dyspnea. This work warrants future investigation into the mechanisms behind our finding of increased large conducting airway luminal area. Discovery of novel components of the pathophysiology of ILD may provide potential targets for pharmaceutical and medical intervention and, as a result, improve clinical outcomes and morbidity in patients with ILD.

## AUTHOR CONTRIBUTIONS

AJM, JWS, CCW, and AHR were involved in conceptualization. AJM, EAO, and AHR were involved in data curation. AJM, JWS, CCW, and AHR were involved in formal analysis. AJM, SZ, JWS, CCW, and AHR were involved in investigation. BTW, MJJ, and AHR were involved in supervision. AJM and SZ were involved in visualization. All authors were involved in writing—original draft. All authors were involved in writing—review and editing.

## FUNDING INFORMATION

The research was supported, in part, by the National Heart, Lung, and Blood Institute (F32HL154320 to JWS; 5R35HL139854 to MJJ) and the Natural Sciences and Engineering Research Council of Canada Postdoctoral Fellowship (AHR).

## CONFLICT OF INTEREST STATEMENT

No conflicts of interest, financial or otherwise, are declared by the authors.

## Supporting information


Appendix S1.

## Data Availability

Analyses and generated datasets that support the current study are not available publicly. The datasets are available from the corresponding author on reasonable request.
