# Supplementary material for: Greater large conducting airway luminal area in adult patients with interstitial lung disease
Source: Physiol Rep. 2025 Sep 24;13(18):e70578. doi: 10.14814/phy2.70578 (PMC12459309; doi:10.14814/phy2.70578)
Supplement: Supplementary file 1 — Appendix S1. [file PHY2-13-e70578-s001.docx]

**Supplementary Appendix**

This appendix has been provided by the authors to give readers additional information about their work.

**Supplement to:** Greater large conducting airway luminal area in patients with interstitial lung disease

**Most Recent Update:** April 07, 2025

**Greater Large Conducting Airway Luminal Area in**

**Patients with Interstitial Lung Disease**

**Supplementary Appendix**

**Contents**

**Supplemental Table S1.** Airway size of men and women previously diagnosed with interstitial lung disease (ILD) and a height- and age-matched control cohort. 3

**Supplemental Table S2.** ILD diagnosis. 4

**Supplemental Figure S1.** Matches chosen using 1:1 nearest neighbor matching algorithm based on height of males (dark blue squares) and females (red circles) with ILD and controls. 5

**Supplemental Table S1.** Airway size of men and women previously diagnosed with interstitial lung disease (ILD) and a height- and age-matched control cohort. Airway size data are the midpoints of each airway. Data are reported as mean ± standard deviation (SD) for normally distributed data and are reported at median (IQR) for non-normally distributed data. Data are compared using separate univariate ANOVAs for normally distributed data and are compared using Kruskal-Wallis tests for non-normally distributed data. *P*-values are reported for between group comparisons (ILD vs. control) for men and women separately.

| **Airway Luminal Size** | **Men** | | |  | **Women** | | |
| --- | --- | --- | --- | --- | --- | --- | --- |
|  | **ILD** | **Control** | ***P*-value** |  | **ILD** | **Control** | ***P*-value** |
| Trachea, mm^2^ | 353 ± 68 | 294 ± 60 | **<0.001** |  | 250 ± 56 | 204 ± 42 | **<0.001** |
| Right main bronchus, mm^2^ | 251 ± 45 | 192 ± 32 | **<0.001** |  | 174 ± 47 | 125 ± 28 | **<0.001** |
| Right upper lobe, mm^2^ | 89 ± 19 | 71 ± 21 | **<0.001** |  | 64 ± 16 | 49 ± 13 | **<0.001** |
| Bronchus intermediate, mm^2^ | 136 ± 28 | 109 ± 24 | **<0.001** |  | 97 ± 23 | 70 ± 19 | **<0.001** |
| Left main bronchus, mm^2^ | 167 ± 34 | 128 ± 27 | **<0.001** |  | 112 ± 32 | 81 ± 21 | **<0.001** |
| Left upper lobe, mm^2^ | 96 (89-104) | 75 (59-91) | **0.001** |  | 74 ± 19 | 56 ± 16 | **<0.001** |
| Left lower lobe, mm^2^ | 65 (52-79) | 50 (37-63) | **<0.001** |  | 56 (49-64) | 39 (32-46) | **<0.001** |

**Supplemental Table S2.** ILD diagnosis

| **Classification** | **N** |
| --- | --- |
| Idiopathic Interstitial Pneumonia | **31** |
| Idiopathic Pulmonary Fibrosis | **27** |
| Hypersensitivity Pneumonitis | **12** |
| CTD-associated ILD | **3** |
| Unclassified ILD | **3** |
| Idiopathic Lymphoid interstitial pneumonia | **2** |
| Scleroderma | **2** |
| Polymyositis-associated ILD | **1** |
| Granulomatous interstitial lung disease | **1** |


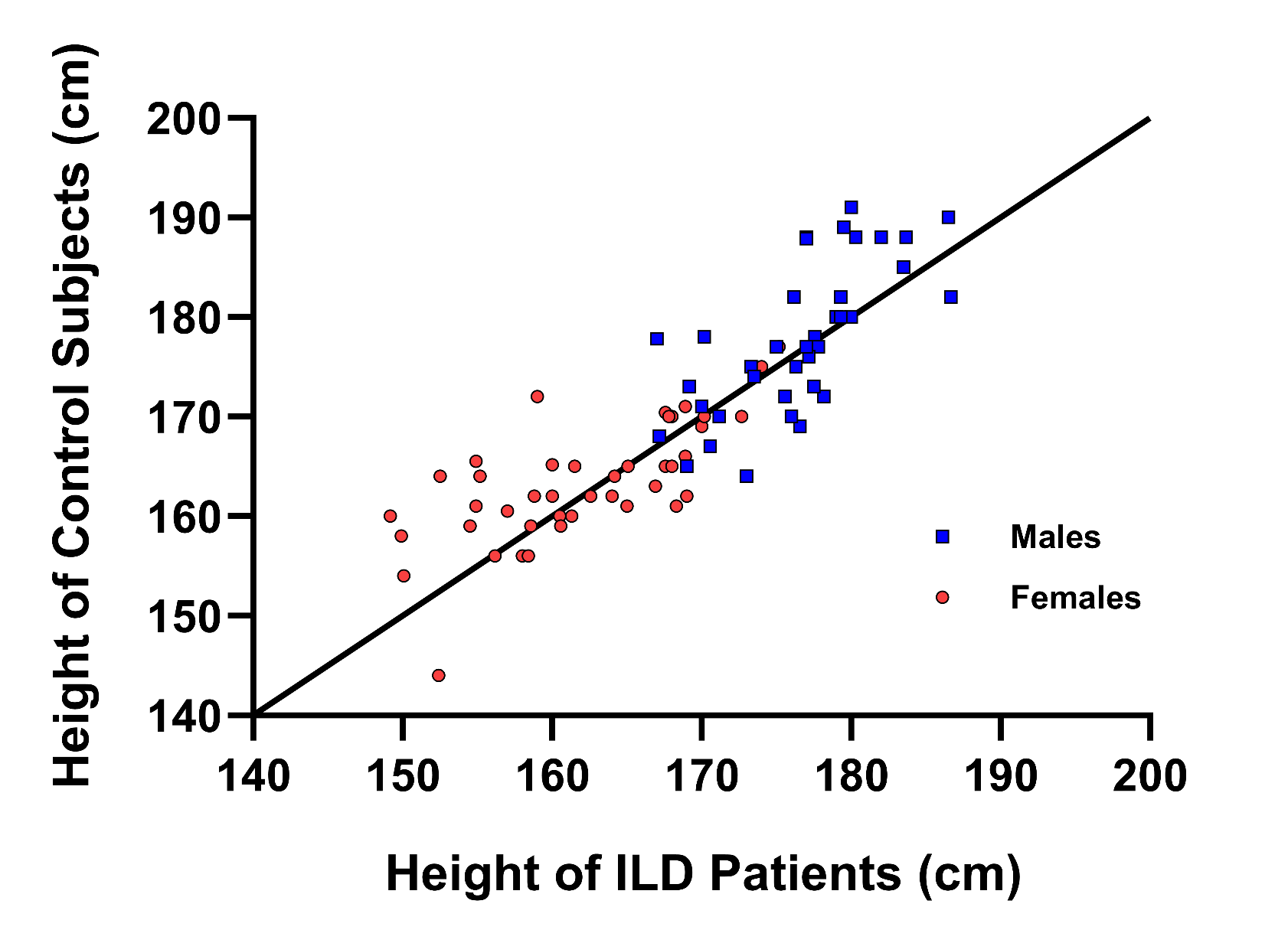
**Supplemental Figure S1.** Matches chosen using 1:1 nearest neighbor matching algorithm based on height of males (dark blue squares) and females (red circles) with ILD and control subjects. Symbols represent height of patients with ILD and control subjects, and the line of identity represents optimal height matching.
